# Deep Learning for Image Watermarking: A Comprehensive Review and Analysis of Techniques, Challenges, and Applications

**DOI:** 10.3390/s26020444

**Published:** 2026-01-09

**Authors:** Marta Bistroń, Jacek M. Żurada, Zbigniew Piotrowski

**Affiliations:** 1 Institute of Communication Systems, Faculty of Electronics, Military University of Technology, 00-908 Warsaw, Poland; zbigniew.piotrowski@wat.edu.pl; 2 Electrical and Computer Engineering, University of Louisville, Louisville, KY 40292, USA; jacek.zurada@louisville.edu

**Keywords:** deep learning, digital watermarking, image watermarking, neural networks, robustness, watermarking algorithms

## Abstract

**Highlights:**

**What are the main findings?**

- Deep learning-based watermarking methods (CNN, GAN, Transformers, and diffusion models) significantly outperform traditional spatial- and frequency-domain techniques in terms of robustness, transparency, and adaptability to modern attack types.
- Emerging architectures such as Vision Transformers, Swin Transformers, and diffusion models introduce new capabilities, notably higher resistance to generative and latent-space attacks, as well as increased watermark capacity.

**What are the implications of the main findings?**

- The rapid evolution of neural network architectures accelerates the development of watermarking systems capable of protecting digital content against increasingly sophisticated threats, including AI-generated manipulations.
- Future watermarking deployments will require optimized, scalable, and computationally efficient deep learning architectures to support real-time applications in cybersecurity, multimedia distribution, IoT systems, and content authenticity verification.

**Abstract:**

The growing demand for digital content protection has significantly increased the importance of image watermarking, particularly in light of the rising vulnerability of multimedia content to unauthorized modifications. In recent years, research has increasingly focused on leveraging deep learning architectures to enhance watermarking performance, addressing challenges related to transparency, robustness, and payload capacity. Numerous deep learning-based watermarking methods have demonstrated superior effectiveness compared to traditional approaches, particularly those based on Convolutional Neural Networks (CNNs), Generative Adversarial Networks (GANs), Transformers, and diffusion models. This paper presents a comprehensive survey of recent developments in both conventional and deep learning-based image watermarking techniques. While traditional methods remain prevalent, deep learning approaches offer notable improvements in embedding and extraction efficiency, particularly when facing complex attacks, including those generated by advanced AI models. Applications in areas such as deepfake detection, cybersecurity, and Internet of Things (IoT) systems highlight the practical significance of these advancements. Despite substantial progress, challenges remain in achieving an optimal balance between invisibility, robustness, and capacity, particularly in high-resolution and real-time scenarios. This study concludes by outlining future research directions toward develop robust, scalable, and efficient deep learning-based watermarking systems capable of addressing emerging threats in digital media environments.

## 1. Introduction

The rapid development of internet and network technologies has led to the widespread digitization of everyday life [1]. Vast amounts of data (text, music, images, video) are processed and shared online on a daily basis. Digital data has become a fundamental resource underlying many social and economic activities. For this reason, one of the key challenges of the 21st century is to balance the protection of digital data with technological and economic advancement [2]. The main threats to digital content security include unauthorized copying and redistribution of content (multimedia piracy) [3,4,5], data manipulation and forgery [6], such as the creation of deepfakes [7], and the privacy-related abuses including unauthorized data usage.

**Figure 1:**
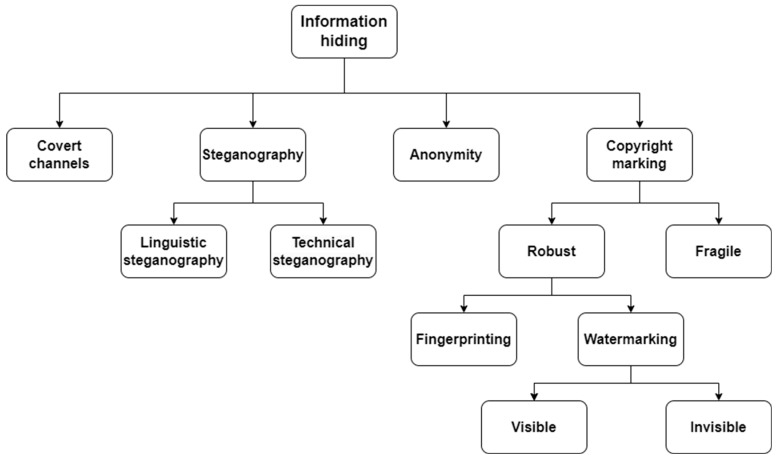
Classification of data hiding techniques based on [8].

To mitigate risks associated with digital data, various protection technologies have been developed, playing a pivotal role in ensuring data security. A classification of data-hiding techniques is presented in Figure 1. Among the mentioned methods, the most widely used are steganography, digital watermarking, and fingerprinting.

Steganography involves the creation of hidden communication channels that enable the transmission of data in a manner imperceptible to third parties. The data is concealed within a carrier, which may include an image, video, audio, or text file [9]. Another form of steganography is network steganography, which focuses on embedding data within transmitted network packets or the communication mechanisms of protocols [10]. The primary objective of steganography is to conceal the very existence of communication. This distinguishes it from cryptography, which protects data through encryption but does not obscure the fact that communication takes place [11]. Digital watermarking focuses on embedding a watermark into a digital carrier, which can be either visible or invisible [12]. Unlike steganography, its purpose is not to conceal the existence of data but, most often, to identify the content owner. A similar technique is fingerprinting, which involves inserting unique markers into each copy of digital content. Both methods frequently rely on comparable technical solutions, with the difference lying in their purpose and scope of application. A watermark is typically embedded in the original content before distribution, whereas a fingerprint serves as a unique marker for each copy, enabling its identification and tracking [13]. Both techniques are often used in combination to provide more comprehensive protection of digital content.

Although steganography, watermarking, and fingerprinting play a crucial role in protecting digital data, they increasingly face sophisticated attacks and the growing complexity of multimedia content. Traditional algorithms often prove insufficient in addressing these issues. An increasingly popular solution is the application of deep learning algorithms, which can automatically learn patterns, adapt to various conditions, and demonstrate resilience to interference, making them effective tools for modern data-hiding techniques.

The subsequent sections of this article will focus exclusively on digital watermarking—its currently employed solutions and future development directions utilizing deep learning techniques. The key contributions of this work are as follows:

- A review and taxonomy of classical watermarking methods for images and video frames.
- A comprehensive overview and taxonomy of deep learning-based image watermarking techniques, highlighting their advantages, limitations, and potential application areas.
- Detailed comparisons of various deep learning architectures (CNNs, GANs, Transformers, and diffusion models) used in watermarking, with particular emphasis on their performance, robustness, and computational complexity.
- A review and comparison of key datasets used for training watermarking algorithms.
- An analysis of future research directions and practical challenges in areas such as deepfake detection, cybersecurity, and applications in IoT systems, with a special focus on the integration of deep learning methods into watermarking solutions.
- A discussion of dataset availability, training strategies, and the role of transparency metrics based on neural networks, as well as specialized robustness metrics tailored to assess the impact of generative and adversarial attacks, providing practical guidelines useful for real-world implementations.

In contrast to previous surveys [14,15] on deep learning-based watermarking, this work adopts a more detailed and practice-oriented perspective, focusing not only on the classification of methods but also on their architectural evolution and practical deployment. Most existing reviews concentrate on conventional deep learning architectures, such as convolutional neural networks (CNNs) and generative adversarial networks (GANs), commonly used in watermarking tasks.

In this review, we frame the development of watermarking techniques through the lens of architectural and design philosophy. Each new model represents an effort to overcome limitations of prior approaches and enhance the overall capacity and robustness of watermarking systems. Accordingly, we incorporate and analyze the latest techniques based on advanced architectures such as Vision Transformers (ViT, Swin Transformer) and diffusion models, which—as discussed in detail later in the paper—demonstrate clear advantages over traditional CNN- and GAN-based solutions, particularly in terms of robustness against attacks and embedding flexibility. Furthermore, this survey goes beyond theoretical discussion to address practical considerations related to real-world implementation. We examine challenges such as computational complexity, data availability, and the evaluation of watermarking performance in terms of both perceptual transparency and resilience to adversarial and generative attacks. This holistic approach aims not only to synthesize the current body of knowledge, but also to facilitate its application in the development and deployment of robust, scalable watermarking systems in practical engineering contexts.

The remainder of this article is organized as follows. Section 2 presents the fundamentals of digital watermarking, providing a detailed description of the general workflow, key paradigms, and commonly used metrics. Section 3 reviews traditional watermarking methods, covering techniques in the spatial, frequency, and hybrid domains. Section 4 focuses on deep learning-based watermarking, discussing various architectures, including CNNs, GANs, Transformers, and diffusion models, with an in-depth comparison of their effectiveness and applications. Section 5 centers on datasets used in watermarking research, detailing available databases, their characteristics, and applications. Section 6 outlines future research directions, emphasizing architectural innovations and application-oriented challenges in areas such as deepfake detection, cybersecurity, and IoT. Section 7 concludes the article with a comprehensive summary, highlighting current challenges and potential solutions for the advancement of watermarking technology in the era of deep learning.

## 2. Fundamentals of Digital Watermarking

### 2.1. Watermarking Workflow

**Figure 2:**
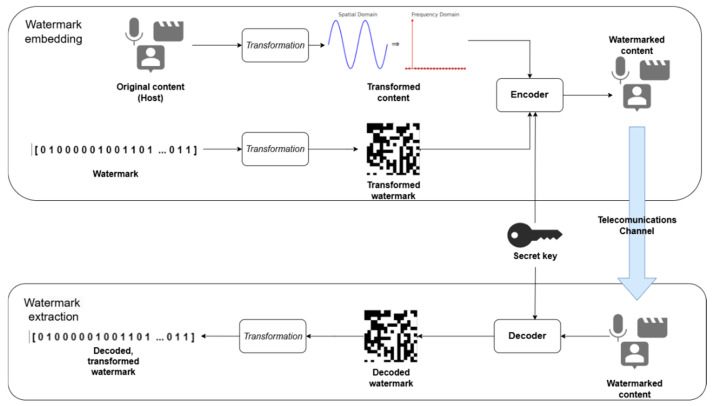
The watermark embedding and extraction scheme, with a secret key controlling the embedding and detection processes.

The watermarking process consists of two fundamental stages: watermark embedding and watermark extraction, as illustrated in Figure 2.

The watermark embedding process begins by transforming the original content (host) into a selected domain (e.g., the frequency domain), where the watermark is inserted. Additional transformations may be applied to further enhance the method’s effectiveness. Optionally, the watermark itself can be transformed depending on the method’s requirements, content type, and intended application. Digital signal processing (DSP) algorithms, by operating on signal parameters such as the phase angle, can embed additional information into the useful signal [16,17], which is particularly valuable in the watermarking of digital objects. During embedding, a chosen algorithm or architecture (encoder) introduces the watermark into the host through minor modifications to its content. The watermarked content is then transformed back into its original domain and transmitted through a telecommunication channel. During transmission and subsequent processing, it may be exposed to intentional or unintentional attacks that could remove or distort the watermark. The extraction process follows a similar approach. A decoding algorithm (decoder), compatible with the embedding method, processes the content in order to retrieve the embedded data. If the watermark underwent transformations or encryption during the embedding stage, inverse transformations are applied to restore it to its original form.

**Figure 3:**
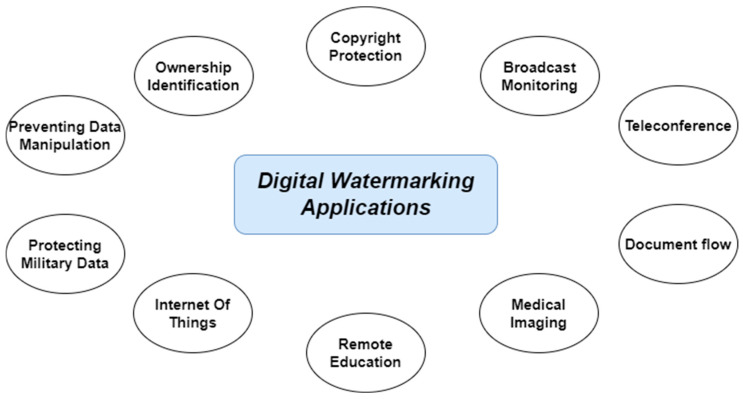
Representative applications of digital watermarking.

The described mechanism forms the foundation for a wide range of practical applications where effective and durable protection of digital content is required. Digital watermarking supports a broad spectrum of use cases depending on the target medium and protection goals. In commercial and legal contexts, it enables copyright protection and ownership verification. In the medical domain, it helps ensure the authenticity and traceability of diagnostic images. In military and IoT systems, watermarking contributes to data integrity and access control. The use cases illustrated in Figure 3 encompass both traditional applications (e.g., document security and media monitoring) and evolving needs in intelligent systems (e.g., teleconferencing and remote education environments).

### 2.2. Watermarking Taxonomy

**Figure 4:**
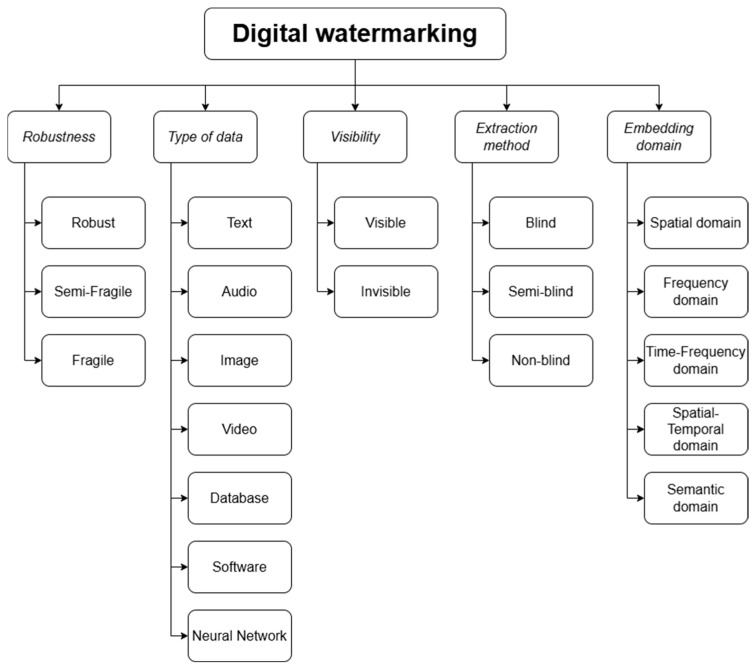
Synthesized taxonomy of digital watermarking methods based on prior literature.

Digital watermarking can be classified according to various criteria. Figure 4 presents a synthesized taxonomy derived from multiple classification schemes reported in the literature, complemented with additional author-defined elements to reflect recent developments and practical perspectives.

Based on their resistance to attacks, digital watermarking methods can be classified into robust, semi-fragile, and fragile categories. In most practical applications, robust methods are used, as they are designed to ensure that the watermark survives typical media processing operations and intentional attacks aimed at damaging or removing the watermark [18]. Semi-fragile methods are usually resistant to processing operations but not to more intensive modifications or deliberate attacks. They are intended for systems where minimal interference with quality is crucial, and intensive data processing is not anticipated. Fragile systems are intentionally designed so that the data is destroyed or damaged with any modification of the carrier. They are used for detecting manipulations and forgeries in the case of highly sensitive data.

The watermark can be embedded in various carriers, the most common being audio data [19,20,21], text [22,23,24], images [25,26,27], and video [28,29]. Due to technological advancements and the need to protect creators in emerging fields, more advanced forms of watermarking have appeared, such as software watermarking [30,31], database watermarking [32,33], neural network model watermarking [34,35], as well as watermarking techniques applied to digital 3D objects [36]. Methods used in these areas include code obfuscation [37], modification of neural network weights [38], and the use of specially prepared trigger sets, which do not affect the model’s functionality but enable the activation of the watermark under specific inputs [39].

Based on visibility, watermarks are classified as visible and invisible. Visible watermarks, such as a company logo, are placed in a way that is noticeable but does not hinder content consumption. In contrast, invisible watermarks are designed to be completely imperceptible to the viewer.

During the watermark extraction procedure, typically only the carrier with the embedded watermark is required; such methods are referred to as blind watermarking. If additional information is necessary for extraction, the methods are classified as semi-blind and non-blind. In semi-blind methods, reference data or a key used during watermark embedding is usually required, such as an attention mask. In non-blind methods, the use of the original data carrier is essential, and the extraction process involves comparing the watermarked data with the original content.

In practical watermarking systems, the use of secret keys is very popular because they play a key role in ensuring security, especially when it is assumed that embedding and extraction algorithms are widely known. The watermark key controls both the embedding and detection processes and is the primary mechanism for preventing unauthorized insertion or extraction of watermarks. Depending on the system design, the same key may be used for both embedding and detection, or separate keys may be used. This key-based security assumption is particularly important in semi-blind watermarking schemes, which dominate real-world applications.

There are two primary domains for watermark embedding: the spatial domain, where the watermark is inserted directly into image pixels or audio samples, and the frequency domain, where the watermark is embedded into the coefficients of a selected transform, allowing, among other benefits, higher resistance to lossy compression. To improve efficiency, hybrid domains are also used, typically combining the advantages of both the spatial and frequency domains [40]. Among the hybrid approaches, the time-frequency domain enables better performance for dynamic signals such as audio or video, while the time-spatial domain, mainly used for video, allows for embedding the watermark both statically and in temporal changes between frames. The semantic domain can be considered a variant of the spatial domain, where watermarks are embedded in areas with specific semantic significance to minimally impact perception.

The remainder of this article focuses on image watermarking methods that ensure robustness and invisibility.

### 2.3. Watermarking Paradigms and Metrics

In the commercial watermarking applications, it is essential for the technology to meet three fundamental paradigms that determine system’s effectiveness and quality: robustness, transparency, and bit capacity. These characteristics are interdependent, making it difficult to improve one criterion without affecting the others.

#### 2.3.1. Transparency

Transparency, or the invisibility of the watermark to the human visual system, is a key feature that often determines whether a given technology will be implemented. It is assessed using both objective metrics, which measure differences between the original carrier and the carrier with the embedded watermark, and subjective metrics, which evaluate content quality based on user perception.

Objective metrics

The most commonly used objective transparency metrics applied to images are listed below:

- MSE (Mean Squared Error)—the average squared error between the pixel values of the original image and the watermarked image.
  (1) $$MSE=\frac{1}{mn}\sum_{0}^{m-1}\sum_{0}^{n-1}{\left\|f\left(i,j\right)-g(i,j)\right\|}^{2},$$

*f*—the matrix data of the original image,
*g*—the matrix data of the watermarked image,
*m*—the number of pixel rows in the images and *i* represents the index of that row,
*n*—the number of pixel columns in the image and *j* represents the index of that column.

- PSNR (Peak Signal-to-Noise Ratio)—measures the ratio of the original image signal to the noise introduced by the watermarking process.

(2) $$PSNR=20{log}_{10}(\frac{{MAX}_{f}}{\sqrt{MSE}}),$$

*MAX*—the maximum possible value of a pixel (e.g., 255 for an 8-bit image).

- SSIM (Structural Similarity Index) [41]—measures the structural similarity between two images by analyzing contrast, brightness, and texture. The values range from 0 to 1, with values closer to 1 indicating higher similarity.

(3) $$SSIM\left(x,y\right)=\frac{\left(2\mu_{x}\mu_{y}+C_{1})(2\sigma_{xy}+C_{2}\right)}{\left(\mu_{x}^{2}+\mu_{y}^{2}+C_{1})(\sigma_{x}^{2}+\sigma_{y}^{2}+C_{2}\right)},$$

*µ_x_, µ_y_*–mean luminance values for X and Y images,
*σ_x_ σ_y_*—standard deviation values for X and Y images,
*σ_xy_*—covariance value between X and Y images,
*C*_1_ *C*_2_—stabilizing constants to prevent division by zero, where

(4) $$C_{1}={\left(K_{1}L\right)}^{2},$$

(5) $$C_{2}={\left(K_{2}L\right)}^{2},$$

*L*—dynamic range of the pixel values (255 for 8-bit grayscale images),
*K*_1_, *K*_2_—small constant, K_1_ ≪ 1 and K_2_ ≪ 1.

- MS-SSIM (Multiscale Structural Similarity Index) [42]—an extension of SSIM that considers multiple spatial scales, calculated through a multi-stage downsampling process.

(6) $$MS\_SSIM\left(x,y\right)=\prod_{j=1}^{M}{\left[{SSIM}_{j}(x,y)\right]}^{\alpha_{j}}\;,$$

*M*—number of scale levels,
*α_j_*—weights (usually α_j_ = 1/M),
${SSIM}_{j}\left(x,y\right)$—SSIM value at the j level.

- VIF (Visual Information Fidelity)—measures the amount of visual information transferred from the original image to the watermarked image using the HVS (Human Visual System) model.

(7) $$I_{0}=\sum {log}_{2}(1+\frac{\sigma_{x_{k}}^{2}}{\sigma_{n_{k}}^{2}}),$$

*I*_0_—the amount of information in the original channel,
$\sigma_{x_{k}}^{2}$—the variance of signal at the *k* level,
$\sigma_{n_{k}}^{2}$—the variance of noise at the *k* level.

(8) $$I_{w}=\sum {log}_{2}(1+\frac{\sigma_{x_{k}}^{2}}{{\sigma_{n_{k}}^{2}+\sigma }_{e_{k}}^{2}}),$$

*I_w_*—the amount of information in the modified channel,
$\sigma_{e_{k}}^{2}$—the variance of error resulting from the difference between the original and the modified image.

(9) $$VIF=\frac{I_{w}}{I_{0}},$$

- FSIM (Feature Similarity Index)—compares key visual features from the perspective of human perception.

(10) $$FSIM\left(x,y\right)=\frac{\sum_{i\in Ω}PC_{m}(i)\cdot S_{L}(i)\cdot S_{P}(i)}{\sum_{i\in Ω}PC_{m}(i)},$$

$PC_{m}(i)$ —phase congruence at a point *i*,
$S_{L}(i)$—luminance similarity function,
$S_{P}(i)$—phase congruence similarity function.

Subjective Metrics

Subjective metrics are used to obtain direct user assessments, where individuals evaluate the quality of an image or video based on their own perception. Testers determine the extent to which content modifications are noticeable and how they affect the overall experience. The conditions and methodology for conducting these tests are thoroughly described in the International Telecommunication Union recommendations, specifically for television images [43] and video materials in lower bandwidth applications such as videoconferencing [44]. However, the methodology can also be successfully applied to static images:

- DSIS (Double Stimulus Impairment Scale)—the method involves comparing two versions of the same material: the original reference version and the processed version. Users view both versions sequentially and then assess the degree of quality degradation in the processed version relative to the original. Ratings are collected on a quality scale from 1 to 5, where 5 indicates that the differences between the original and the modified content are imperceptible, and 1 signifies that the content quality has significantly deteriorated and is unacceptable.
- DSCQS (Double Stimulus Continuous Quality Scale)—users are presented with both the original and the modified versions of the content, but they are not explicitly informed which is the original or the modified version. Similar to DSIS, users evaluate the content quality; however, the lack of clarity regarding which material has been altered provides a more objective assessment from the perspective of human perception.
- Paired Comparison Test—users are shown two versions of the content: the original and the modified, without indicating which one has been altered. Participants evaluate which version they believe has higher quality or whether they can notice any differences.

AI-Based Perceptual Metrics

With the development of deep learning techniques, modern perceptual metrics utilizing neural networks have emerged, offering a better reflection of human image perception compared to classical similarity metrics [45]. Selected deep learning-based metrics used to evaluate the transparency of watermarking systems include:

- LPIPS (Learned Perceptual Image Patch Similarity) [46]—this method employs convolutional networks trained on large datasets to measure perceptual differences between images. Metric values close to 0 indicate smaller differences and higher transparency of the method.
- DISTS (Deep Image Structure and Texture Similarity) [47]—a metric that combines texture and structure analysis using deep features from neural networks, providing improved assessment of images with complex details.
- PieAPP (Perceptual Image-Error Assessment through Pairwise Preference) [48]—a trained model that predicts image quality based on user preferences by evaluating pairs of images.

#### 2.3.2. Robustness

Watermark robustness refers to the system’s ability to retain the watermark within the carrier even after processing operations such as compression, filtering, scaling, or intentional attacks aimed at removing the watermark. The assessment of robustness involves comparing the quality of the original watermark embedded in the carrier with the watermark extracted after processing operations. Among the robustness evaluation metrics, the following are distinguished:

- BER (Bit Error Rate)—measures the percentage of bits that have been incorrectly extracted from the embedded watermark compared to the original watermark.

(11) BER=1N∑i=1N|wi−wi^|,

*w_i_*—value of the *i*-th bit in the original watermark,
wi^—value of the *i*-th bit in the extracted watermark,
*N*—total number of bits.

- NC (Normalized Correlation)—a metric that measures the similarity between the original and the extracted watermark. A value close to 1 indicates high resistance to attacks.

(12) NC=∑i=1Nwi·wi^∑i=1Nwi2·∑i=1Nwi^2,

- ASR (Attack Success Rate)—it represents the percentage of successful attempts to weaken or remove the watermark as a result of adversarial attacks [49].
- RGA (Robustness Against Generative Attacks)—a metric evaluating the system’s resistance to attacks utilizing generative models. It analyzes the extent to which the watermark remains intact after being processed by these models [50].
- APT (Adversarial Perturbation Tolerance)—defines the minimum level of perturbations introduced by adversarial attacks necessary to successfully remove or distort the watermark. A higher APT value indicates greater system robustness [51].

#### 2.3.3. Capacity

Watermark capacity refers to the amount of information that can be embedded in the carrier while maintaining adequate transparency and robustness. Below are the most commonly used capacity metrics:

- Payload Capacity—measures the number of watermark bits relative to the given carrier.

(13) $$P=\frac{N_{bit}}{N_{host}},$$

*N_bit_*—number of bits embedded as watermark,
*N_host_*—number of host units (pixels for images, seconds for video or audio).

- Embedding Capacity Efficiency—measures the efficiency with which the system utilizes the carrier’s space for embedding the watermark, taking into account the impact on quality and robustness.

(14) $$ECE=\frac{N_{bit}}{N_{max}}\cdot 100\%,$$

*N_max_*—maximum capacity of the host, i.e., how many bits can be embedded before the quality of the content is noticeably degraded.

### 2.4. Attacks on Watermarking Systems

In practical applications, watermarking systems are exposed to various processing operations and deliberate attempts to remove or distort the embedded watermark. The previously described metrics measure how well a watermarking system withstands attempts to destroy or alter the watermark. However, different types of attacks can cause diverse distortions to the carrier and the embedded watermark, making the analysis of their effects crucial for a comprehensive system evaluation. Below is an overview of the main types of attacks along with their impact on carrier quality and watermark integrity, as summarized in Table 1. The attacks presented in the table are divided into three main categories: untargeted attacks including standard multimedia processing operations, targeted attacks; and deep learning attacks. The last category is distinguished as a separate group due to its dynamic development, high effectiveness, and the difficulty in counteracting such attacks.

Deep learning-based attacks, especially those exploiting latent space regeneration (e.g., via diffusion models or VAEs), tend to be significantly more effective than traditional signal-domain manipulations. The high effectiveness of these attacks stems from the fundamental operating principles of modern generative models. Diffusion models and variational autoencoders learn compact latent representations that capture the semantic structure of the image while discarding high-frequency or weakly correlated signal components. As a result, watermarks embedded in the pixel or frequency domain are often treated as noise during the generative reconstruction process and are not preserved when the image is regenerated from the latent space, yielding visually faithful content devoid of the original watermark.

Unlike traditional signal-domain attacks, which typically apply local distortions or global transformations to the original signal, latent-space regeneration reconstructs the image from learned data distributions. Consequently, synchronization-based and redundancy-based watermarking schemes-effective against compression or geometric attacks-become ineffective when the original content is replaced by a newly synthesized instance that preserves perceptual quality but not the embedded watermark.

This highlights the growing need for DL-robust watermarking strategies that are explicitly designed to counter generative and latent-space attacks. Recent research approaches include embedding watermarks directly into latent representations, integrating watermark-preservation constraints into the training objectives of diffusion or GAN-based generators, and developing joint generation–watermarking frameworks in which watermark survival becomes an inherent property of the synthesis process. Such strategies aim to shift watermarking from a post-processing operation to an integral component of content generation, thereby improving resistance to regeneration-based attacks.

## 3. Traditional Image Watermarking Methods

Traditional watermarking methods are primarily based on modifying the carrier data in the spatial and the frequency domain, as well as on hybrid-domain approaches that aim to combine the advantages of both, as extensively discussed in the classical watermarking literature [54].

### 3.1. Spatial Domain

Spatial domain methods are among the simplest and oldest techniques in digital watermarking. They rely on direct modification of pixel values in images or video frames without prior transformation into another domain. A well-known example is the LSB (Least Significant Bit) technique proposed by Turner [55], in which the least significant bits of pixels are modified to hide information. These methods are characterized by high capacity and low complexity but relatively low resistance to attacks. One of the first digital watermarking approaches utilizing this technique was proposed in 1994 [56]. Over the years, the method has undergone numerous modifications. In [57], the authors proposed using the third and fourth LSBs to improve data embedding. Subsequently, in [58], a combination of the LSB method with binary value inversion of the watermark was introduced, enhancing the method’s transparency. Arya and Saharan [59] increased both the robustness and transparency of the LSB method by generating the watermark image from the host image and securing it with a key derived from the same source. In [60], the authors modified the traditional approach by introducing a hashing mechanism for the watermark before embedding, which improved resistance to attacks. Another approach combines the LSB method with edge detection techniques [61] to identify suitable regions for watermark embedding and additionally encrypt the watermark to increase the method’s security.

The LSB method can also be applied to video signals when watermarking is performed on a frame-by-frame without considering temporal dependencies. In [62], the authors used LSB to embed the watermark into selected video frames and employed an FPGA architecture to accelerate the embedding and extraction processes in real-time. Similarly, in [63], the watermark was embedded into specific video frames using the LSB algorithm, with the frame selection based on histogram analysis.

Among other methods based on pixel value modification, the following can be distinguished: Pixel Value Differencing (PVD) [64,65], the Patchwork algorithm [66,67,68], Singular Value Decomposition (SVD) [69,70], and the Arnold transformation [71,72].

### 3.2. Frequency Domain

In frequency-domain watermark embedding methods, the content is transformed using a selected transform, after which the watermark is embedded into the transform coefficients. Once embedded, the watermarked content is transformed back into the original domain. This approach generally exhibits higher resistance to content processing operations, such as compression, scaling, and deliberate watermark attacks. In the case of images, watermark embedding most commonly utilizes the Discrete Cosine Transform (DCT) [73,74,75,76,77,78], the Discrete Fourier Transform (DFT) [79,80,81,82], and the Discrete Wavelet Transform (DWT) [83,84,85,86]. Transform-based methods offer numerous advantages over spatial domain watermarking approaches. DCT-based solutions are known for their high resistance to compression. DWT, owing to its multilevel analysis capabilities, increases resistance to basic processing operations, while DFT enables watermark embedding in frequency ranges that are less susceptible to modifications, making the watermark more robust against geometric transformations. Combinations of different transforms enable the integration of their individual strengths, resulting in solutions with greater robustness and higher transparency, such as DCT–DWT [87,88,89,90], DFT–DCT [91,92,93], DFT–DWT [94,95,96], and DFT–DCT–DWT [97].

Similar to the spatial domain approaches, it is possible to apply one-dimensional or two-dimensional transforms, including video signals, provided that watermarking is performed on individual frames without considering temporal context. This approach has been proposed in the following publications for the transforms: DCT [98,99,100,101,102], DWT [103,104,105,106], 1D-DFT [107], 2D-DFT [108,109], as well as their combinations, which aim to improve the method’s efficiency [110,111,112,113].

### 3.3. Hybrid Domains

Typical hybrid domains, such as the time-frequency and the time-spatial domain, are mainly applied in video signal watermarking. In the case of images, the semantic domain can be used, which is classified as hybrid since it combines elements from both the spatial and frequency domains. However, its key feature is content-level analysis at the semantic level. The watermark is embedded in selected areas of the image or video that are significant from a content perspective. This approach enables the watermark to be hidden in regions that are perceptually or semantically important to the user, making the watermarking process both more resistant to manipulation attempts and less noticeable. Semantic domain methods rely on various image processing techniques, such as segmentation, edge detection, and object detection [114].

### 3.4. Summary

**Figure 5:**
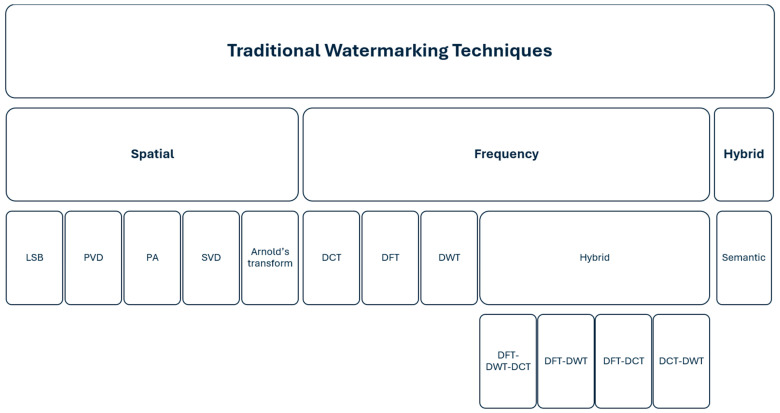
Taxonomy of traditional watermarking algorithms for images and video frames. Spatial-domain methods include LSB [56,57,58,59,60,61,62,63], PVD [64,65], PA [66,67,68], SVD [69,70], and Arnold’s transform [71,72]. Frequency-domain methods include DCT [73,74,75,76,77,78,98,99,100,101,102], DFT [79,80,81,82,107,108,109], and DWT [83,84,85,86,103,104,105,106]. Hybrid approaches combining DFT–DWT–DCT [97,113], DFT–DWT [94,95,96], DFT–DCT [91,92,93], and DCT–DWT [87,88,89,90,91,92,110,111,112] are also shown. Semantic watermarking methods are presented in [114].

In summary, the classification of traditional watermarking methods for images and video frames is presented in Figure 5.

## 4. Deep Learning-Based Watermarking

In recent years, the rapid development of deep learning-based technologies has revolutionized many areas of science, including digital watermarking. Unlike traditional methods, which rely on manually designed features, deep learning algorithms learn optimal data representations, enabling more efficient, robust, and invisible watermark embedding.

Deep learning is a subset of machine learning that utilizes multi-layer neural networks (deep neural networks) [115,116]. Due to their architecture and ability to automatically recognize and learn patterns, DL algorithms can learn to be robust to various types of targeted and untargeted attacks by appropriately defining loss functions. Additionally, neural networks are highly efficient in solving problems that require scalability. This is due to the hierarchical structure of feature learning, the ability to process in parallel, and support for multi-dimensional data [117], making neural networks very effective even in watermarking high-resolution images and videos, such as 4K resolution.

### 4.1. Deep Learning Architectures Used in Image Watermarking

**Figure 6:**
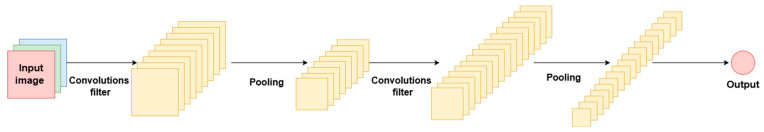
Convolutional Neural Network schema.

The fundamental architectures used in image watermarking are convolutional neural networks. These are a type of deep neural networks specifically designed for analyzing data with a matrix-like structure, such as images or video frames, which has ledthem to dominate the processing of such data for years [118]. The network consists of convolutional blocks, which include convolutional layers, activation layers, and pooling layers. The key component are the convolutional layers, which consist of filters whose primary function is to extract features from images, enabling edge detection, texture identification, and the recognition of more complex patterns. In watermarking, CNNs are utilized in watermark encoder and decoder algorithms. The convolutional filters learn to modify input images in such a way that the watermark is embedded, ensuring that the watermark is both transparent and resilient to attacks. The primary advantage of such CNN-based solutions is their simplicity of implementation and flexibility in adapting to different input data and watermarks. The general block diagram of a convolutional network is shown in Figure 6.

**Figure 7:**
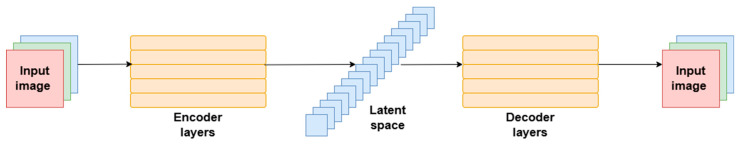
Autoencoder schema.

Although CNNs form the basis of many watermarking algorithms, to achieve algorithms that meet more stringent requirements in terms of robustness and transparency, it is also necessary to use other architectures. Autoencoders provide an optimized approach to data encoding and decoding data. Their architecture allows data to be transformed into a different (hidden) space, followed by data reconstruction [119]. An autoencoder consists of two parts—Figure 7:

- Encoder, which transforms the input data into a lower-dimensional representation, with the goal of reducing the data size while capturing the most important features of the input,
- Decoder, which reconstructs the data based on the representation by applying reverse transformations to those used in the encoder.

Autoencoders are trained in an unsupervised manner, minimizing the difference between the input and output data by using an appropriately selected cost function [120,121,122]. The architecture is a natural choice for watermarking tasks because the encoder–decoder structure mirrors the process of embedding and extracting a watermark.

**Figure 8:**
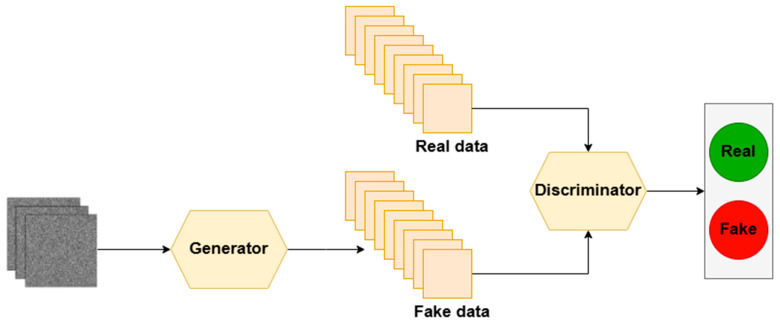
Generative Adversarial Networks schema.

Another approach for creating more efficient watermarking algorithms is the use of Generative Adversarial Networks. This architecture relies on two competing neural networks [123]—Figure 8:

- Generator, based on provided features or random noise, learns to generate new data,
- Discriminator attempts to distinguish between real data and data generated by the generator.

In watermarking applications, an extension of this concept is typically used, namely DCGAN (Deep Convolutional Generative Adversarial Networks)—a GAN architecture built on convolutional networks [124]. The generator learns to embed the watermark into the provided data in an invisible manner, while the discriminator, acting as a critic, evaluates whether the watermark has been properly hidden. Due to their operating principles, GANs enables the development of algorithms characterized by high transparency and are also easily adaptable to different types of data.

**Figure 9:**
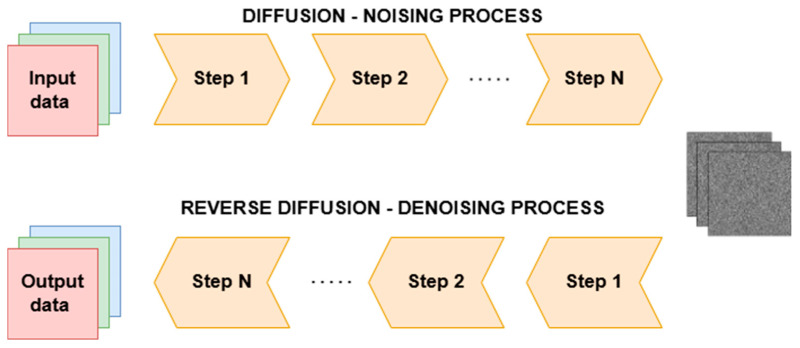
Diffusion model schema.

The main drawback of GANs is their instability, as the competition between the generator and the discriminator makes them quite difficult to train. A potential solution to these problems lies in the use of diffusion models. These are generative structures that, through the use of a reverse diffusion process, learn to generate or reconstruct data from random noise [125,126]. The input data is iteratively noised in a controlled manner until it becomes completely random noise. Then, the model learns the reverse process—recovering the original data from the noisy data. The conceptual diagram of the model is shown in Figure 9. Diffusion models are not yet widely adopted in watermarking solutions, but due to their noise removal mechanism, they can be highly effective in extracting watermarks even after various types of attacks.

**Figure 10:**
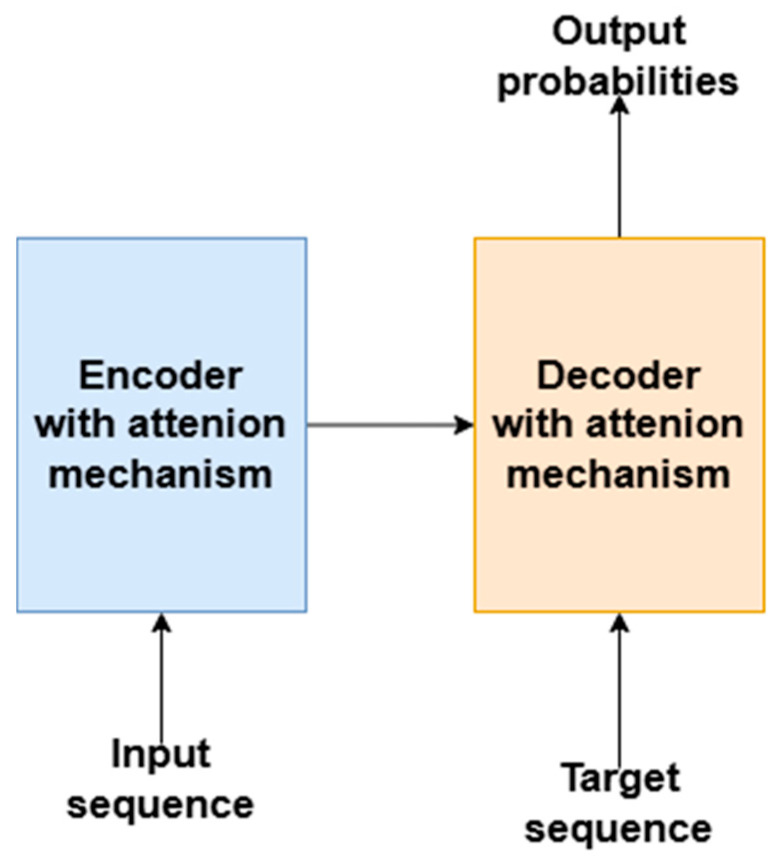
Transformer schema.

Currently, Transformer models are increasingly being used, surpassing the efficiency of their predecessors. A Transformer architecture consists of two components: an encoder with an attention mechanism that transforms the data into an internal representation, and a decoder with an attention mechanism that, based on the input representation and the target sequence, predicts the next sequence elements with a given probability [127]—Figure 10.

The central element of Transformers is the attention mechanism, which enables the capture of dependencies between components of the input sequence, regardless of their relative distance. This mechanism, known as self-attention, analyzes how each element of the sequence is related to the others. In image watermarking applications, a specialized version called spatial attention [128] is used, which focuses on spatial relationships in the input data, such as between pixels or pixel blocks in images. The spatial attention mechanism helps identify key areas for analysis, such as the optimal location for watermark embedding.

For image processing applications, a dedicated variant of the Transformer, the Vision Transformer (ViT), was developed. Unlike traditional convolutional neural networks (CNNs), ViTs divide the image into smaller fragments (called patches), which are then transformed into vectors and processed through the self-attention mechanism. This approach allows the model to capture global dependencies within the image. While Vision Transformers [129] can achieve higher performance compared to CNNs, they require a larger amount of training data to be effective.

### 4.2. Overview of Deep Learning-Based Image Watermarking Algorithms

Given the diversity of visual data, varying resolutions, and applications requirements, different solutions are employed, each tailored to specific problems. These solutions have been described and classified in literature reviews [130,131,132].

The first attempts to apply deep learning in image watermarking emerged around 2017. One of the initial approaches was based on a CNN that operates similarly to an autoencoder [133]. Two independent CNNs generate two image sets, created as a codebook, which are then permuted using cryptographic keys. For each bit of the watermark, an appropriate pair of codebook codes is selected and embedded into the image. This method enables the embedding of a 64 × 64-pixel watermark into a 128 × 128-pixel image, offering resistance to common image processing attacks and JPEG compression while ensuring a high level of security due to the use of cryptographic keys. In [134], the authors proposed a blind watermarking method based on CNNs using an end-to-end approach in which embedding and extraction processes are optimized together within a single architecture. The neural network embeds a 1-bit watermark in each sub-block of the image with dimensions 8 × 8 pixels. Subsequently, selected geometric attacks and signal processing operations are simulated to enhance the algorithm’s robustness.

In subsequent work, architectures based on the use of two or three main modules—a watermark encoder, decoder, and, optionally, a module simulating attacks on the watermark—became dominant. In [135], the authors proposed an architecture that enables embedding a watermark in the form of an audio file into an image. Two neural networks were utilized: WM Network and Similarity Network. The WM Network consists of the Encoder and Decoder modules, which map the watermark using LSTM, and Embedder and Extractor, which are based on convolutional layers. Similarity Network compares the original watermark with the extracted one, allowing the evaluation of the method’s effectiveness. A similar approach was adopted in [136]. This is a blind algorithm based on CNNs, designed to extract the watermark from images captured using mobile phones. The architecture includes components responsible for mapping and demapping the watermark, as well as its embedding and extraction, all composed of convolutional layers. A key element of the algorithm is the Invariance Layer, which affects the algorithm’s resistance to attacks. The module is based on fully connected layers and allows for dispersing information across different parts of the image.

In [137], the authors also used a CNN-based encoder and decoder, but during the preprocessing of the host image, they applied the Wavelet Transform and an additional convolutional network responsible for watermark preprocessing. Moreover, the authors implemented an attack simulator to enhance the algorithm’s robustness against basic image processing operations. A similar architecture, based on an encoder and decoder composed of convolutional layers along with an attack simulator, was implemented in [138]. The authors employed DWT before embedding the watermark and IDWT (Inverse Discrete Wavelet Transform) after embedding to restore the image to its original domain. The watermark is in the form of a binary image with dimensions of 32 × 32 pixels, eliminating the need for an additional watermark processing block.

Lu et al. [139] also used the Wavelet Transform but incorporated it within the embedding algorithms. As the encoder, they employed the U-Net autoencoder architecture [140], in which the central convolutional and deconvolutional blocks were replaced with DWT and IDWT blocks. In the decoder, convolutional layers and DWT blocks were implemented. Between the encoder and decoder blocks, an attack simulation block inspired by StegaStamp was introduced. StegaStamp [141] is a solution that enables the embedding of hyperlinks into images. The encoder uses a convolutional network, also similar to U-Net. A key component of the system is the distortion simulation block, which, unlike most watermarking techniques, enhances the algorithm’s resistance to distortions introduced during the printing of images. This feature allows the method to be used in real-world physical scenarios, such as printed photographs or billboards.

In order to increase the transparency of the algorithm, the authors in [142] utilized an autoencoder architecture in both the watermark encoder and decoder. In the encoding algorithm, the watermark embedding occurs in the latent space, after which the image is scaled back to its original resolution. In the decoder, a denoising autoencoder is first used to reduce the effects of noise and other distortions (if present). Subsequently, two encoders are employed to extract the watermark based on both the original image and the image with the embedded watermark. The network training is conducted in two stages.

In subsequent approaches, the encoder–decoder architecture was enhanced with an attention mechanism. Dasgupta and Zhong [143] proposed a solution utilizing the multi-head cross-attention mechanism (MHA) [127], which enables the model to learn mutual dependencies between two different data sequences, in this case, between the watermark and the host image. Additionally, the authors employed representation learning in an invariant domain using a triplet loss function [144], which optimizes the distances between images containing the same watermark content (anchor and positive) while maximizing the differences between them and a negative image (any other image). This approach improves the robustness and the model’s ability to learn the watermarking pattern.

The second group of algorithms consists of GAN-based approaches, where a discriminator module is added to the encoder and decoder blocks. The watermark encoder functions as a data generator, while the discriminator acts as a critic, assessing the visibility of the watermark. The first such solution was the HiDDeN architecture [145] proposed by Zhu et al. It is an end-to-end solution based on three CNNs. The encoder embeds a bit sequence into the host image; the watermarked image is then processed through a distortion layer, after which the decoder extracts the watermark. The discriminator verifies whether the watermarked image is sufficiently similar to the original image, allowing the method to achieve high transparency.

Wen and Aydore proposed an improvement to this approach by developing the ROMark algorithm [146]. The authors utilized the HiDDeN architecture, introducing min-max optimization, which involves optimizing losses under the worst-case scenario. This is achieved through the implementation of a dynamic noise layer that iteratively generates the most challenging possible distortions. Additionally, the range of applied distortions was expanded, and gradient propagation was improved to facilitate more efficient network training. The HiDDeN algorithm was also used in [147], where the authors extended the distortion module by adding a rotation layer and an additive noise layer. They also modified the loss function, which enabled a better trade-off between robustness and transparency. In [148], the authors similarly focused on optimizing the learning process proposed in [145]. They introduced a two-stage training process, training the encoder and decoder for the base model, and training separate decoders for different types of distortions in the second stage to better enhance the watermark’s resistance to various types of attacks.

A completely different GAN-based model was proposed in [149]. The authors utilized the Inverse Gradient Attention (IGA) mechanism, which dynamically identifies image pixels most resistant to distortions and assigns them higher weights during the message-hiding process. Additionally, the encoder module enables the compression of binary messages into real numbers, allowing the embedding of a larger number of bits (256 bits) without affecting image transparency. A similar approach, employing a different algorithm, was presented by Hao et al. [150]. The authors applied attention mechanisms in both the encoder (generator) and decoder to identify areas most resistant to disturbances and focus on them during watermark embedding. The implemented discriminator not only serves as a critic but also evaluates the visual quality of images and supports robustness optimization. A dynamic disturbance layer was also incorporated to simulate multiple attacks simultaneously. Attention mechanisms were further utilized in [151]. The ARWGAN model employs an attention mechanism in the encoder to generate an attention mask, enabling the placement of the watermark in the most optimal image regions. Additionally, a Feature Fusion Module was used to extract image features and leverage them to enhance robustness. The authors also implemented a Noise Subnetwork to simulate various types of distortions. In [152], the authors applied a GAN-LSTM structure combined with the Adaptive Gannet Optimization algorithm, which, similar to previous solutions, enables the selection of optimal locations for watermark embedding. The watermark undergoes preprocessing using DWT and Schur decomposition and is subsequently chaotically encrypted. Incorporating the LSTM architecture into the algorithm allows for better management of the watermark extraction process, improving both accuracy and robustness.

The previously described attention-based solutions enabled the dynamic identification of optimal image regions for watermark embedding. An extension of this concept involves the use of more advanced architectures, such as Transformers, which bring new capabilities to watermarking-related challenges. In [153], the authors implemented Transformers for both text processing and visual Transformers for image feature extraction as part of the proposed text embedding algorithm. The text is encoded into a vector representation with dimensions 16 × 64 using a Transformer, while the encoder and decoder are based on the ViT architecture. To improve algorithm robustness, noise was added to the encoded text representation, and common image distortions were applied to the watermarked image. In [154], a ViT was also employed for image authentication, manipulation detection, and image recovery. The source image undergoes preprocessing using several transforms: Discrete Wavelet Transform, Integer Wavelet Transform, Schur decomposition, and Curvelet Transform, the latter enabling the identification of high-entropy areas suitable for watermark embedding. The model generates encoded feature maps to serve as watermarks. Additionally, an authentication key is generated using Singular Value Decomposition.

Liu et al. [155] proposed a two-stage approach for watermark embedding using Transformers. The input images are transformed using DWT, enabling decomposition into frequency coefficients, with the watermark embedded in the low-frequency components to increase robustness. The attention module stores information about both low and high frequencies, enhancing the stability and accuracy of image reconstruction. As part of the two-stage embedding procedure, the encoder and decoder are first trained to embed and extract the watermark, and then a reversible information embedding procedure is introduced based on the first stage (freezing the encoder weights) to increase robustness. In [156], a transformer was combined with a GAN structure through the implementation of a discriminator that supports the transparency of the image with the embedded watermark. In the encoder, self-attention-based preprocessing was used to expand the watermark features and increase embedding efficiency by evenly distributing the watermark information across the image. Additionally, a Feature Enhancement Module was utilized to identify relationships between the image and the watermark using cross-attention, and a Soft Fusion Module, responsible for the final fusion of image and watermark features based on self-attention and cross-attention. The solution also includes a Noise Layer responsible for simulating disturbances and increasing robustness.

In [157,158], the authors employed the Swin Transformer architecture [159], which was designed for image processing and is characterized by better global information flow and lower computational complexity than ViT. In [157], the Swin Transformer was combined with CNN. The watermark encoder is based on CNN and blocks Squeeze-and-Excitation (SE), which support the extraction of important features. In the decoder, CNN was innovatively used to analyze local features, Swin Transformer, which allows for hierarchical feature processing and improves global feature representation, and the Identity module, which, through residual connections, facilitates feature extraction. Additionally, a Multi-scale Attentional Feature Fusion module (MA-FFM) was implemented to iteratively combine global and local features. In [158], the END (Encoder-Noise-Decoder) structure was used. The watermark encoder is based on the U-Net architecture, supplemented with a Locally-Channel Enhanced Swin Transformer Block, which utilizes window-based self-attention, and a Frequency-Enhanced Transformer Block, which applies an attention mechanism in the frequency domain using the cosine transform. The window-based self-attention mechanism involves dividing the image into smaller areas (windows) and analyzing dependencies only within the window area rather than globally across the entire image, followed by window shifting. The decoder structure is similar to the encoder but uses additional attention mechanisms to precisely recover the watermark.

The next significant step in the field of watermarking was the use of diffusion models, which allow for even greater resistance to disturbances and better control over the structure of embedded information. Initial research on watermarking in the context of diffusion models focused primarily on the detection and marking of images generated by these models [160,161,162]. In the subsequent phase, diffusion algorithms themselves began to be used for embedding and extracting watermarks. One of the first such approaches is the ZoDiac algorithm [163], described by Zhang et al., which utilizes the Stable Diffusion model [164] to embed watermarks in the latent space of the image. The watermark is embedded into the frequency coefficients of the Fourier transform, making it more resistant to attacks and manipulations, including generative attacks (Stable Diffusion-based removal attacks). Stable Diffusion is a text-to-image model developed for text-to-image generation. ZoDiac uses a pre-trained model with a denoising step of 50 to reconstruct the image in the latent space, which reduces the time and resources required to deploy the system.

The WaterDiff method proposed in [165] is also based on a pre-trained diffusion model. The host image is transformed by two separate encoder modules into a latent feature vector of high-level features and a matrix of low-level features. The watermark is then embedded into the coefficients of the wavelet transform, and the final watermarked image is reconstructed into its original form using the diffusion model. The extraction process proceeds analogously and relies on the use of the pre-trained probabilistic model. A distinctive feature of this approach is its very high watermark capacity of 1 bit per pixel (bpp) and the flexible ability to adjust the frequency subspace in which the watermark is to be hidden.

In [166], the SuperMark algorithm is described, which is also based on the use of a pre-trained diffusion model, but dedicated to super-resolution (SR). The watermark embedding process involves transforming the input image to a lower resolution compatible with the SR model input, embedding the watermark in the latent space through Gaussian Shading, and denoising and reconstructing the image with the embedded watermark back to its original resolution. During watermark extraction, the image is again scaled to a lower resolution and transformed into the latent space using variational autoencoder. The use of the diffusion model allows the reconstruction of the original Gaussian noise, from which the watermark bits are extracted.

### 4.3. Summary of Deep Learning-Based Image Watermarking Algorithms

**Figure 11:**
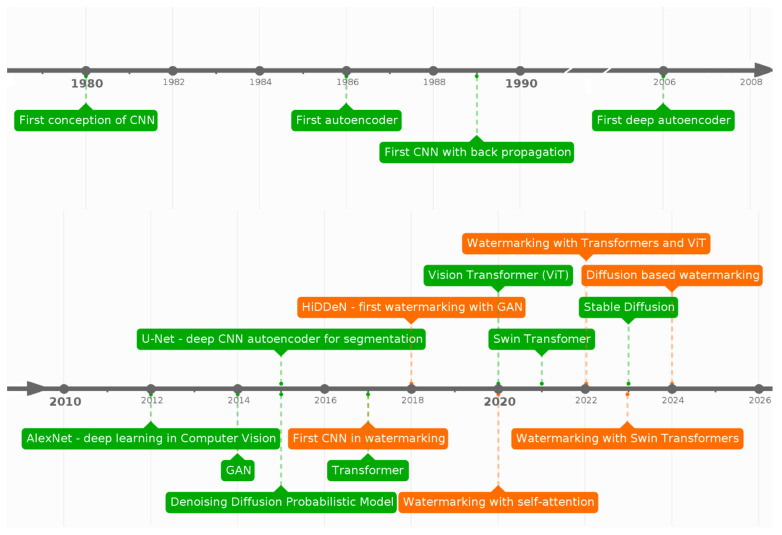
Timeline of development of deep learning-based image watermarking methods.

The development of image watermarking techniques based on deep learning has followed the evolution of neural network architectures. Figure 11 illustrates a historical timeline of key deep learning architectures (marked in green) alongside their applications in watermarking (marked in orange). The watermarking methods referenced here were described in the previous subsection. The emergence of convolutional networks (1980) and their subsequent popularization (2012) revolutionized image processing. Furthermore, the introduction of deep autoencoders (2006) further expanded the capabilities of neural networks, leading to advanced image encoding techniques such as U-Net (2015).

The first application of CNN in watermarking appeared in 2017, and by 2018, the groundbreaking HiDDeN model was developed—the first deep learning-based watermarking method utilizing GANs. Simultaneously, the introduction of Transformers (2017) and their improvements dedicated to image processing (ViT, 2020; Swin Transformer, 2021) paved the way for watermarking applications leveraging the attention mechanism. In 2022, watermarking methods based on ViT emerged, followed by those utilizing the Swin Transformer in 2023.

The latest trend in image watermarking is the use of diffusion models, which, although introduced in 2015, gained significant popularity in 2023 following the publication of the Stable Diffusion model, capable of text-to-image conversion. Diffusion-based methods have the potential to significantly enhance resistance to adversarial attacks (particularly the increasingly common generative attacks) by embedding watermarks in the latent space.

It is worth noting the decreasing time between the development of a new architecture and its application in watermarking. In the early stages, technologies such as convolutional networks or autoencoders were used for watermarking only several years after their inception. However, with the advancement of deep learning, increased availability of pre-trained models, and improved computational resources, this time has significantly decreased: 4 years for the GAN architecture, and 2 years for Transformers and Swin Transformers. The most dynamic progress can be observed with Stable Diffusion, which was adapted for image watermarking within approximately one year after its release through the ZoDiac architecture [163]. This trend suggests that new technologies are being implemented in watermarking almost immediately after their development, driven by the growing availability of pre-trained models and the increasing demand for more resilient methods to protect digital content.

The evolution of image watermarking methods has led to the development of techniques with diverse properties and applications. Table 2 presents a comparison of key methods described in the previous subsection. Due to significant inconsistencies in experimental conditions across the surveyed studies—including variations in image resolution, watermark capacity, attack types, and evaluation metrics—it is not feasible to provide a standardized quantitative comparison of metrics such as PSNR, SSIM, or BER. As a result, Table 2 focuses on qualitative attributes that remain relatively comparable across works, such as architectural design, general watermark capacity, and robustness to commonly reported attacks.

The architectural changes in watermarking methods can be seen not merely as technical refinements, but as reflections of an evolving design philosophy—one that seeks to address the three fundamental challenges of image watermarking: embedding capacity, perceptual transparency, and robustness against attacks. The comparative analysis across CNNs, GANs, Transformers, and diffusion models clearly reflects how different design strategies prioritize or balance these criteria. The first approaches, based on CNNs in encoder–decoder architectures [134] or autoencoders [133] offer simplicity and computational efficiency, but may lack resistance to more complex manipulations. GAN-based solutions improved watermark concealment and realism, at the cost of stability and training complexity. Transformer-based models introduced greater capacity and robustness through attention mechanisms, while diffusion models provide enhanced resilience against generative attacks by embedding in the latent space. With the advancement of technology, the capacity of embedded watermarks has significantly increased, reaching values of bpp = 1 in some methods [165]. Additionally, a growing number of modern watermarking methods are being designed to be resolution-independent, allowing for effective application in HD (high definition) and 4K materials [167,168]. Thus, the architectural perspective adopted in this review implicitly captures the evolution of trade-offs between robustness, invisibility, and capacity—a key concern in watermarking system design.

## 5. Datasets for Image Watermarking

In principle, any image dataset, whether labeled or unlabeled, can be used to train a watermark embedding and extraction algorithm. However, in the case of deep learning-based methods, the quality and diversity of the training data play a crucial role in determining the model’s effectiveness. A well-chosen dataset enables better model generalization, which enhances resistance to attacks and improves watermarking effectiveness under various conditions [169]. Below are the key criteria that a dataset should meet to be suitable for training a watermarking algorithm:

- High resolution—Modern watermarking methods should be tested not only on standard images of 128 × 128 or 256 × 256 pixels but also on high-resolution images such as HD (1080p) and 4K, which is essential for practical applications such as multimedia content protection [170];
- Content diversity—The dataset should include both real-world images (landscapes, faces, animals, objects, vehicles) and graphics or textures. This is particularly important for methods utilizing attention mechanisms, which rely on contextual relationships between image elements [129];
- Open access—Free and open access to data facilitates research replication and the comparison of different solutions’ effectiveness, forming the foundation for reliable evaluation of watermarking methods [171];
- High visual quality—Images should be artifact-free, clear, and detailed, allowing for precise evaluation of watermark transparency and its impact on the visual quality of the image after embedding and extraction [172];
- No lossy compression—Lossless formats (e.g., TIFF or PNG) are preferred to avoid artifacts resulting from lossy compression (e.g., JPEG), ensuring a reliable assessment of the watermarking method’s resistance to image degradation [173];
- Synthetic and real images—With the growing popularity of generative models such as DALL-E [174] and Midjourney, there is an increasing need to watermark content generated by artificial intelligence. Therefore, the dataset should include both real and synthetic images to ensure algorithm effectiveness in both contexts [175].

Considering the above features when selecting a dataset is crucial for optimizing the effectiveness of watermarking methods and their resistance to various attack scenarios. Based on these criteria, the datasets used can be divided into four categories:

- Benchmark datasets—These are classical image sets widely used in image processing and deep learning research. They are characterized by standard resolutions, usually 256 × 256 pixels, and a high diversity of content, enabling versatile usage;
- High-resolution datasets—These include images with HD and 4K resolutions. Originally intended for training super-resolution algorithms, they are now successfully used to evaluate watermarking effectiveness in real-world applications where high visual quality is essential;
- Synthetic datasets—Comprising images generated by AI models, these datasets feature visual characteristics that differ from those of real-world images. As a result, models trained on such datasets may require adapted watermarking methods to perform effectively;
- Specialized datasets—These include images from specific fields, such as medicine, geoinformatics, security, or digital documents, where watermarking plays a key role in ensuring data integrity and authenticity. Such images are characterized by a high level of detail and specific visual features, often necessitating tailored watermarking approaches.

Table 3 presents an overview of the most commonly used datasets in image watermarking research. Key attributes are included, such as resolution, number of images, and thematic content. For each dataset, its main limitations and typical applications in watermarking experiments are also indicated. This aims to facilitate the evaluation of their suitability for various research objectives, such as testing embedding capacity, robustness, or imperceptibility. All listed datasets are publicly available resources.

Most datasets used in image watermarking research were not originally created for watermarking purposes but were primarily developed for classification [176,177,178,185,189], identification [188,190], or image segmentation [177,179]. Nevertheless, due to their diversity, high quality, and wide availability, they are successfully adapted to evaluate watermarking methods in terms of both resistance to attacks and transparency verification. In particular, benchmark datasets and high-resolution datasets enable the evaluation of watermarking methods under realistic conditions, while synthetic datasets are playing an increasingly significant role in watermarking AI-generated content.

## 6. New Research Directions and Challenges in Image Watermarking

The dynamic development of deep learning-based watermarking methods has made image watermarking not only more efficient and effective but also more technically and implementationally complex. Traditional approaches based on spatial and frequency domain transforms are gradually losing their effectiveness in the face of the increasing number of generative attacks and the growing need to ensure resistance to various visual distortions. As a result, watermarking is encountering new challenges and development directions, which define current research priorities and determine the future of this technology in the context of digital content protection [191].

### 6.1. Key Challenges in Implementing Image Watermarking Systems

The practical implementation of deep learning-based image watermarking solutions involves a range of technological, performance, and legal challenges. Methods utilizing artificial intelligence algorithms are significantly more computationally complex than their classical counterparts, which rely on much simpler mathematical algorithms [192]. Their implementation requires advanced models such as autoencoders, convolutional networks, generative networks, or transformers, all characterized by a high number of parameters and the necessity to operate on large datasets [193]. This issue becomes particularly significant when dealing with high-resolution images (e.g., 4K or 8K), where both the embedding and extraction processes demand substantial computational power.

In addition to computational complexity, a fundamental challenge in the practical design of watermarking systems remains the inherent trade-off between three key objectives: robustness, perceptual transparency, and embedding capacity. Achieving a balance between these aspects is non-trivial, as improvements in one dimension often lead to compromises in another. For example, highly robust watermarking methods—especially those based on diffusion models [141]—tend to introduce higher computational costs and longer processing times, while increasing capacity may impact transparency. In particular, diffusion-based approaches, though capable of superior robustness against generative and latent-space attacks, remain difficult to optimize for real-time or resource-constrained applications due to their iterative nature and substantial hardware demands. As a result, ongoing research increasingly focuses not only on model efficiency but also on understanding how architectural choices influence this robustness–transparency–capacity trade-off in practical deployment scenarios.

This trade-off becomes particularly evident in the recent architectures, where excessive computational load and prolonged data processing time constitute a major barrier to real-time deployment. In diffusion-based watermarking methods [194], despite their high robustness and transparency, impose substantial hardware and memory requirements, limiting their applicability in resource-constrained environments.

From a deployment perspective, it is also important to distinguish between training and inference costs in deep learning–based watermarking systems. While training phases—particularly for transformer- and diffusion-based models—are typically performed offline and can leverage high-performance computing resources, inference-time requirements directly affect system feasibility in real-world applications. Large model sizes, memory footprints, and iterative generation processes may limit the applicability of such approaches in real-time, embedded, or resource-constrained environments. Consequently, practical watermarking deployments often prioritize architectures with moderate model size and predictable inference latency, even at the cost of reduced robustness against advanced attacks.

From a quantitative perspective, deep learning architectures used in watermarking differ substantially in scale and computational cost. CNN-based watermarking models typically consist of only a few million parameters and allow single-pass inference, making them suitable for real-time processing on standard GPUs or even high-end embedded devices. In contrast, transformer-based architectures often require tens to hundreds of millions of parameters, while diffusion-based watermarking systems involve iterative generation processes with dozens or hundreds of diffusion steps, resulting in significantly higher inference latency and memory consumption [195]. These order-of-magnitude differences, rather than exact numerical values, are the dominant factor determining deployment feasibility across application scenarios [196].

In the face of increasing computational demands, there is a need to develop techniques that reduce the complexity of deep learning models in the field of watermarking, where embedding effectiveness, processing time, and resource efficiency are crucial. One approach is the use of lightweight architectures designed to require fewer parameters and computational operations while maintaining acceptable processing quality [197]. Examples include models like MobileNet [198] and SqueezeNet [199], which, thanks to a reduced number of filters and innovative convolutional layers, can efficiently operate even on mobile devices.

Another popular strategy is weight pruning, which involves eliminating model parameters that have minimal impact on the output. This reduces the number of insignificant connections in the neural network while maintaining comparable accuracy [200,201]. An alternative method is quantization, which does not eliminate parameters but reduces their bit precision, thereby decreasing model size and accelerating computations [202].

In the context of diffusion models, modifications are gradually emerging to reduce the number of iterations required to achieve high-quality results. Examples include methods that integrate the inference process into the training phase for joint optimization [203] or those aimed at accelerating sampling [204].

Transfer learning is also widely used, allowing previously trained models to be adapted to new domains or tasks, thus shortening training time and reducing data requirements. In diffusion-based learning, pretrained models are utilized to denoise images with embedded watermarks in the latent space, ensuring high transparency and bypassing the costly process of training a model from scratch [163].

Despite the implementation of numerous techniques to limit computational complexity, the scalability of watermarking systems remains a challenge. With the growing number of users, the diversity and volume of image data, and the demand for real-time processing, traditional solutions may prove inadequate [205]. Designing and deploying scalable methods require both suitable hardware architectures and efficient distributed algorithms capable of dynamically managing computational and memory resources [206].

Cloud infrastructure is one of the most popular solutions for improving the scalability of watermarking systems. The cloud enables, among other things, the centralization of deep learning models [207], which significantly simplifies the process of updating and deploying new versions without user-side intervention. Additionally, serverless services allow for automatic scaling in response to changing loads [208].

Another approach is edge computing, which shifts part of the computational operations closer to the data source—directly onto mobile devices or edge servers [209]. This is particularly important for real-time watermarking systems, as it minimizes latency during live transmission, facilitates watermarking of images in IoT systems, and enables fast multimedia content authentication [210]. Edge computing reduces network load since images do not need to be transmitted to a centralized server and also enhances data security [211], by allowing local watermarking without leaving the user’s device.

In the training process itself, federated learning is commonly used, enabling deep learning models to be trained using distributed datasets without needing to centralize data on a single server [212]. In watermarking, this approach is especially valuable when data is sensitive or too large to consolidate easily.

From a scalability perspective, federated learning enables efficient utilization of the computational resources of multiple devices simultaneously, reducing reliance on central servers and allowing the training process to scale to thousands of nodes [213]. This enables the parallelization of training process without increasing network load and mitigates bottlenecks associated with transmitting large datasets. An added advantage is the model’s adaptability to local data, which can improve watermarking effectiveness in specific conditions and for particular end devices. Federated learning is also used in training diffusion models, significantly reducing the number of parameters while maintaining high output image quality [214].

Ensuring scalability and efficiency in deep learning-based watermarking systems requires a multidimensional approach that combines architecture optimization, effective resource management, and technology adaptation to specific conditions. The solutions described demonstrate significant potential in reducing computational costs, improving response times, and enhancing data processing security. However, their effectiveness depends on multiple factors, with the greatest efficiency often achieved through a synergistic combination of several techniques. Integrated approaches like Edge Intelligence [215], which combine AI, edge computing, and federated learning, are already opening new possibilities for broad watermarking applications in resource-constrained environments and real-time systems.

### 6.2. Research Directions for Methods and Algorithms

The dynamic development of deep learning in watermarking opens new research directions focused on designing architectures aimed at improving the robustness, efficiency, and transparency of watermarking systems. As shown by the literature review conducted in Chapter 4, two groups of models have begun to dominate recent research: Transformers (Vision Transformer and Swin Transformer) and diffusion models. Both approaches offer an intriguing alternative to previously used solutions, namelyCNNs [216] and generative adversarial networks (GANs) [217].

From a critical standpoint, recent advances in deep learning-based watermarking reveal that the choice of network architecture directly determines not only robustness and transparency but also vulnerability to specific classes of attacks and the feasibility of real-world deployment. Consequently, CNN-, GAN-, Transformer-, and diffusion-based approaches should be viewed as complementary rather than competing solutions, each addressing different design constraints and threat models.

Transformer-based architectures, utilizing various attention mechanism variants, are increasingly applied to diverse image processing tasks [218]. ViT and its improved variant, Swin Transformer, introduce fundamental changes to how images are processed. Unlike traditional CNNs, which analyze images locally, Transformers capture global dependencies between different parts of an image. Swin Transformers further enhance this by introducing a sliding window mechanism, enabling hierarchical image processing and improving both computational efficiency and the ability to analyze local and global features [159].

Diffusion models are generative probabilistic models where image generation (or other processing operations) is performed through iterative noise addition and removal. Unlike GAN models, which often face convergence issues, diffusion model training is much more stable and yields higher quality generated images [115,125,219].

Table 4 and Table 5 present a comparative overview of the key features of CNNs and transformer-based architectures, as well as diffusion models and GANs, with a focus on their applications in image watermarking.

The analysis of the presented data indicates that further research on visual transformers in the context of watermarking should primarily focus on optimizing computational complexity to enhance their potential in real-time systems. Although architectures such as ViT and the Swin Transformer demonstrate high effectiveness in watermark embedding [153,154,157,158], their intensive computational requirements limit practical deployment possibilities. Therefore, an important research direction is the development of lightweight and more efficient versions of transformers capable of effectively operating on end devices such as smartphones or surveillance cameras [220,221,222].

Due to the simplicity and good efficiency of CNNs, combining transformers with convolutional networks in the form of hybrid architectures is a valuable approach. These models, merging the local precision of CNNs with the global context of transformers, can improve watermark durability and resilience while maintaining an acceptable level of computational complexity [223]. With the growing popularity of generative attacks, research should also focus on increasing watermark resistance to such threats [224]. A significant challenge lies in better understanding how the self-attention mechanism affects the placement and durability of the watermark in the image and how it can be used to embed better-hidden yet higher-capacity watermarks [225].

For diffusion models, research mainly focuses on developing technologies that reduce the number of diffusion steps without degrading the quality of the generated images [226]. Traditional models, such as Denoising Diffusion Probabilistic Model or Stable Diffusion, while providing high-quality watermark embedding [163,165,166], are characterized by long generation times, hindering their application in real-time and low-latency environments. In response, efforts are being made to develop accelerated sampling methods and to utilize latent diffusion models, which operate in the latent space [227], significantly shortening watermark embedding time. This approach not only improves watermark resistance against subsequent manipulations but also enables more controlled and precise digital content watermarking [164].

Research in this area also focuses on how the watermark can survive various transformations, such as lossy compression, scaling, and attacks using generative models, which are becoming increasingly common. Additionally, a key development direction involves designing hybrid architectures combining diffusion models with transformers, leveraging the global understanding of images provided by the self-attention mechanism and the stable generation process offered by diffusion [228,229]. Researchers are also exploring methods combining modern neural network architectures with quantum technology [230].

Taken together, the comparisons presented in Table 4 and Table 5 highlight a fundamental trade-off in deep learning–based watermarking. Architectures that provide the highest robustness against generative and latent-space attacks—such as diffusion models and large transformer-based solutions- are currently the most computationally demanding and the least suitable for real-time or resource-constrained deployment scenarios. In contrast, CNN-based and hybrid CNN-Transformer approaches, while offering lower resistance to advanced generative attacks, remain more practical for time-critical and embedded applications due to their reduced computational complexity.

Overall, current research clearly indicates that no single deep learning architecture can simultaneously satisfy all watermarking requirements, including robustness, transparency, capacity, and computational efficiency. As a result, future research increasingly points toward adaptive and hybrid solutions, in which architectural choices are guided by specific application scenarios, threat models, and deployment constraints rather than by the pursuit of a universal, one-size-fits-all watermarking framework.

### 6.3. Application-Oriented Research in Watermarking

Contemporary watermarking is increasingly expanding beyond traditional intellectual property protection [231]. With the use of deep learning, modern watermarking systems are being applied in many key areas, including the identification of AI-generated content, cybersecurity, monitoring systems, and IoT. Below are the current and future applications of watermarking that may dominate this field in the coming years.

#### 6.3.1. Watermarking in Identification of Deepfakes

The rapid development of generative techniques has made the identification and counteraction of so-called deepfakes a key challenge in digital security [232]. Fake multimedia content, generated by artificial intelligence, is becoming increasingly realistic and difficult to distinguish from authentic materials, posing serious threats in the realms of politics, security, and privacy [233].

The use of watermarking techniques is playing an increasingly important role in the recognition of AI-generated content [234], including deepfakes detection. Watermarking algorithms can be employed to embed invisible markers during the image generation process, enabling subsequent verification of whether a given material was synthetically generated. An example of such an approach is SynthID [235], developed by Google, which integrates invisible watermarks into AI-generated content to facilitate their identification. However, ensuring the resilience of these markers against attempts at removal or modification is crucial for effectively combating disinformation on the Internet. Research must also consider techniques that are resistant to common deepfake attacks and operations, such as face alterations, voice modulation, or background modifications.

An important emerging research area is the embedding of metadata in deepfakes. By embedding source information or generation timestamps directly into multimedia content, it becomes possible to detect manipulations and track the origin of materials. Standards such as the Content Authenticity Initiative (CAI) and the Coalition for Content Provenance and Authenticity (C2PA) promote this approach, enabling the embedding of metadata in images and other media to verify their authenticity. The implementation of such standards can significantly aid in the fight against disinformation and facilitate the identification of AI-generated content.

#### 6.3.2. Watermarking in Cybersecurity

In the context of cybersecurity, there is a growing number of applications utilizing watermarking technologies in various ways—often referred to as Cyber Watermarking [236]. A highly promising development direction is the integration of modern watermarking techniques with blockchain technology [237]. Combining these two solutions enables the creation of systems that not only mark digital content but also store cryptographic hashes of the content in a decentralized database [238]. This allows any user to verify the authenticity of multimedia files without concerns about unauthorized manipulation within the chain.

Watermarking is also gaining popularity as an authentication mechanism in both commercial and governmental applications. Embedding digital signatures into images and other documents can serve to confirm their authenticity and prevent unauthorized modifications [239]. Research in this area mainly focuses on increasing watermark resilience against compression, format conversions, and content edits that may occur during data transmission or archiving.

#### 6.3.3. Watermarking in Monitoring and IoT Systems

The dynamic development of Internet of Things technologies and video surveillance systems has opened new perspectives for the application of watermarking, extending beyond traditional multimedia content protection. Modern surveillance systems, integral to smart cities and advanced security infrastructures, rely on the credibility of collected video data. In this context, watermarking algorithms play a crucial role in ensuring the integrity and authenticity of recordings, enabling rapid identification of visual materials and detection of manipulation attempts [240].

One of the most important applications of watermarking in surveillance systems is the verification of recording authenticity for criminal investigations and legal proceedings. Recordings containing an embedded watermark can serve as reliable evidence in court cases, confirming that the material has not been altered since its capture. When data is transmitted over networks or stored long-term, watermarks enable material tracking, allowing any attempt to replace or delete portions of the footage to be quickly detected.

Beyond traditional surveillance systems, watermarking is gaining significance in IoT ecosystems, which include a wide range of image-capturing devices such as drones, wearable cameras, and systems in autonomous vehicles [241]. In such cases, watermarking serves not only to identify the data source and control access but also to verify recording authenticity in real time.

One of the key challenges associated with the use of watermarking in monitoring systems is ensuring resistance to varying environmental conditions. Recordings can be susceptible to distortions caused by lighting changes, weather conditions, or recording angles.

## 7. Conclusions

The rapid development of deep learning technologies in recent years has significantly impacted the field of digital image watermarking, offering new possibilities for improving transparency, robustness, and embedding capacity of watermarking systems. This article provides a comprehensive review of both traditional watermarking methods and the latest deep learning-based approaches, covering architectures such as convolutional neural networks, generative adversarial networks, Vision Transformers, Swin Transformers, and diffusion models. The conducted analysis shows that, while deep learning-based methods offer significant advantages in terms of embedding effectiveness and attack resistance, they also present challenges related to high computational requirements, the need for large datasets, and architectural complexity.

The analysis indicates that the latest architectures —particularly transformers and diffusion models, offer a promising balance between transparency and robustness in watermarking systems. Compared to older neural network architectures, these models can effectively conceal watermarks without noticeably degrading image quality, while demonstrating high resilience against generative attacks, including those performed using advanced deep learning models. This is particularly important in the context of rapidly developing generative technologies, which pose an increasing threat to the integrity of digital content. These trends indicate that transformers and diffusion models playing an increasingly significant role in watermarking research in the coming years. However, it is important to emphasize that other architectures, such as CNNs and GANs, will not be entirely phased out. On the contrary, there is already a significant rise in the popularity of hybrid solutions that combine the strengths of different models—for example, systems integrating transformers with convolutional networks or transformers with diffusion models. Additionally, there is growing interest in supporting classical neural methods with quantum technologies, which may open new perspectives for enhancing performance and increasing the robustness of watermarking systems.

Overall, while deep learning-based watermarking methods have greatly expanded the possibilities for digital content protection, the challenges associated with their practical implementation and the dynamic evolution of generative technologies make further research in this area essential. Moreover, technological changes and an expanding range of applications clearly signal a sift away from an era in which watermarking was primarily associated with copyright protection. With the rapid development of new use cases—such as cybersecurity, Internet of Things (IoT), authentication systems, and multimedia forgery detection—watermarking is becoming crucial for ensuring data integrity, authenticity, and security. Thanks to these new possibilities, watermarking could evolve into a fundamental component of authorization systems and become one of the main tools for content origin identification and credibility verification in the digital ecosystem of the future.

## Figures and Tables

**Table 1:** Types of attacks on watermarking systems.

Group of Attacks	Type of Attacks	Description	Examples of Operations	Effect on the Watermark
Untargeted attacks	-	Attacks resulting from routine processing of the media, with no intention of removing the watermark, but which may affect its integrity.	Lossy compression (JPEG, MPEG), scaling, filtering.	Partial loss or distortion of the watermark.
Targeted attacks	General	Intentional manipulation of the media to remove, distort, or weaken the watermark.	Rotate, scale, change resolution.	Total or partial loss of the watermark.
Targeted attacks	Statistical attacks	Modifications using statistical analysis of the media to identify and remove the watermark.	Histogram attack, frequency distribution analysis, autocorrelation attack.	Removal of the watermark without significant changes in the perception of the media.
Targeted attacks	Sensitivity attacks	Minimal modifications to the media that do not affect the visual quality, but destroy the watermark.	Bit depth reduction, delicate pixel changes.	Distortion or complete loss of the watermark without visible changes in the media.
Targeted attacks	Destructive compression	Aggressive compression to remove the watermark by extremely reducing the media data.	High-loss JPEG compression.	Significant loss of data, complete destruction of the watermark, degradation of media quality.
Targeted attacks	Geometry attacks	Manipulations in the spatial structure of the media that distort the position of the watermark.	Rotation, translation, change of proportions.	Disturbance of the watermark position, loss of synchronization.
Deep learning-based attacks	Generative attacks	Use of generative models to regenerate media content and remove embedded watermark.	Image inpainting, deepfake generation, AI-based restoration.	Complete removal of the watermark without perceptual changes.
Deep learning-based attacks	Adversarial attacks	Modifications generated by neural networks to fool detection systems and weaken watermark extraction.	Adversarial noise, gradient-based attacks (FGSM, PGD).	Degradation or undetectability of the watermark.
Deep learning-based attacks	Neural network removal	Use of DL models trained to detect and remove watermarks.	CNN-based watermark removal, encoder–decoder architectures.	High probability of the watermark elimination with minimal distortion.
Deep learning-based attacks	Content replacement	Media content is regenerated using deep learning models to overwrite or bypass the watermark layer.	GAN-based texture replacement, style transfer techniques.	Loss or severe weakening of the watermark.
Deep learning-based attacks	Latent-space attacks	Attacks that exploit generative models (diffusion or VAE-based) to regenerate content in the latent space, effectively removing embedded watermarks.	Stable Diffusion regeneration, DERO [52], VAE sampling attacks [53].	Total removal of the watermark, especially in latent-domain watermarking schemes.

**Table 2:** Summary of watermarking algorithms based on deep learning.

Year	Reference	Architecture and Technology	Watermark Capacity	Host Image Resolution	Watermark Robustness
2017	[133]	CNN as autoencoder	64 × 64 pixels	128 × 128 pixels	Noise, cropping, JPEG compression, rotation
2017	[134]	CNN with residual blocks	4096 bits	512 × 512 pixels	Affine transform, cropping, JPEG compression, filtering, rotation, rescaling
2021	[135]	CNN and LSTM for audio mapping	8192 audio samples	128 × 128 pixels	Not implemented
2021	[136]	CNN and fully connected Invariance Layer	32 × 32 pixels/1024 bits	128 × 128 pixels	Noise, cropping, blur, JPEG compression
2023	[137]	CNN with DWT as preprocessing	256 bits	256 × 256 pixels	Noise, dropout, JPEG compression
2023	[138]	CNN with DWT as preprocessing	32 × 32 pixels/1024 bits	512 × 512 pixels	Noise, sharpening, smoothing, dropout, JPEG compression
2022	[139]	CNN autoencoder with DWT and IDWT blocks	50–700 bits	400 × 400 pixels	Perspective warp, motion, blur, noise, color manipulation, JPEG compression
2020	[141]	CNN autoencoder	100 bits	400 × 400 pixels	Perspective warp, camera misalignment, blur, color distortion, noise, JPEG compression
2023	[142]	CNN autoencoder and CNN denoising autoencoder	32 × 32 pixels/1024 bits	128 × 128 pixels	Noise, rotation, JPEG compression
2023	[143]	CNN with MHA in Invariant Domain	8 × 8 pixels/64 bits	128 × 128 pixels	Horizontal flip, blur, solarization, brightness adjustment, contrast variation, hue and saturation modulation
2018	[145]	GAN	30 bits	128 × 128 pixels (training) 512 × 512 pixels (testing)	JPEG compression, blur, cropping, dropout
2019	[146]	GAN with min-max optimization	30 bits	128 × 128 pixels	Cropping, cropout, dropout, blur, JPEG compression and combinations
2020	[147]	GAN	64 bits	512 × 512 pixels	Rotation, JPEG compression, noise, cropping, blur, brightness adjustment
2021	[148]	GAN	64 bits	256 × 256 pixels	JPEG compression, rotation, noise, blur cropping, brightness and contrast adjustment, color inversion
2020	[149]	GAN and IGA	256 bits	256 × 256 pixels	Cropping, dropout, JPEG compression, resizing
2020	[150]	GAN and attention	30 bits	64 × 64 pixels	Cropping, cropout, blur, flip, JPEG compression
2023	[151]	attention module, GAN, feature fusion	30 bits	128 × 128 pixels	Cropping, dropout, blur, JPEG compression, resizing
2024	[152]	GAN-LSTM, Adaptive Gannet Optimization	256 × 256 to 1024 × 1024 pixels	256 × 256 to 1024 × 1024 pixels	Noise, median filtering, blur, JPEG compression, cropping, rotation, scaling
2024	[153]	Transformer and ViT	16-word segments	224 × 224 pixels	JPEG compression, noise, rotation, cropping
2023	[154]	ViT	128 bits	256 × 256 pixels	Noise, median filtering, rotation, scaling
2023	[155]	Transformer with DWT preprocessing	binary image 24 × 24 pixels	96 × 96 pixels	Median and gaussian filtering, noise, SPN, JPEG compression, rotation, cropping, scaling
2023	[156]	Transformer, GAN	36 to 100 bits	128 × 128 pixels	Noise, cropout, dropout, JPEG compression, affine transformation
2023	[157]	Swin Transformer, CNN, MA-FFM, Identity module	64 bits	128 × 128 pixels	Cropping, noise, dropout, gaussian and median filtering, JPEG compression
2024	[158]	Swin Transformer with DCT attention block	64 bits	128 × 128 pixels	Cropout, dropout, rotation, scaling, affine transform
2024	[163]	Stable Diffusion	32 bits	64 × 64 × 4 (latent)	JPEG compression, rotation, noise, blur, generative attacks
2024	[165]	Diffusion Probabilistic Model	16384 bits	128 × 128 pixels	JPEG compression, regeneration attacks
2024	[166]	Diffusion Probabilistic Model	32 bits	512 × 512 pixels	JPEG compression, blur, noise, cropping, brightness adjustment, adaptive attacks

**Table 3:** Overview of commonly used datasets in image watermarking research.

Name	Category	Number of Images	Resolution [Pixels]	Type	Limitations/ Notes	Reference
ImageNet	Benchmark	14 million	~224 × 224 to 256 × 256 (resized)	Animals, vehicles, plants, tools	Large scale, requires preprocessing, good for robustness tests.	[176]
COCO	Benchmark	330,000	640 × 480	People, vehicles, food, animals	Moderate resolution, suitable for general-training.	[177]
CIFAR-10/CIFAR-100	Benchmark	60,000/100,000	32 × 32	Animals, vehicles	Very low resolution, not suitable for perceptual metrics, useful for capacity tests.	[178]
Pascal VOC	Benchmark	21,000	500 × 375	Animals, vehicles, people	Limited scale and resolution, used in simple robustness evaluations.	[179]
BOSSbase	Benchmark	10,000	512 × 512	Grayscale natural images	Designed for steganalysis, great for statistical robustness tests.	[180]
DIV2K	High resolution	1000	~2K (e.g., 2040 × 1080)	Landscape, buildings, architecture,	High quality, ideal for transparency tests.	[181]
Flickr2K	High resolution	2650	~2K (e.g., 2040 × 1350)	Natural photos: portraits, landscapes,	Unprocessed, variable quality, useful for perceptual metrics.	[181]
LIU4K	High resolution	2100	4K (3840 × 2160)	Different background and objects	High resolution, good for visual quality and real-world simulations.	[182]
UHD4K	High resolution	5000+	4K (3840 × 2160)	Satellite images, films, urban scenes	Very high resolution, good for high-end use cases.	[183]
UHD8K	High resolution	2966	8K (7680 × 4320)	Satellite images, films, urban scenes	Extremely high resolution, useful for stress testing.	[183]
LAION-5B	Synthetic	5.85 billion	from 256 × 256 to 4K	Images paired with text prompts (mixture of real and AI-generated)	Unprocessed and noisy, not ideal for reproducible benchmarking.	[184]
CIFAKE	Synthetic	120,000	32 × 32	Real images from CIFAR 10 and synthetic images	Low resolution, designed for deepfake detection benchmarks.	[185]
ArtiFact	Synthetic	1.5 million	from 256 × 256 to 1024 × 1024	People, animals, vehicles, artworks	Moderate resolution, good for testing synthetic distortions.	[186]
ImagiNet	Synthetic	200,000	from 256 × 256 to 2K	Photos, painting	Well-balanced synthetic content, useful for hybrid real/synthetic training.	[187]
NIH Chest X-ray	Specialized (medical)	112,000	1024 × 1024	Chest X-rays	Suitable for medical robustness/embedding studies.	[188]
EuroSAT	Specialized (satellite images)	27,000	64 × 64	Satellite images: forests, urban areas, fields	Low resolution, useful for satellite-specific tests.	[189]
LFW (Labeled Faces in the Wild)	Specialized (faces)	13,000	250 × 250	Facial photos in natural conditions	Standard for face datasets, useful for privacy, detection, and watermarking on identity data.	[190]

**Table 4:** Comparison of key features of Vision Transformer, Swin Transformer, and CNN in the context of watermarking.

Feature	CNN	ViT	Swin Transformer
Feature processing method	Local (by convolutional filters)	Global (by self-attention)	Local and global (by shifted windows)
Resistance to traditional attacks	Medium	High	High
Resistance to generative attacks	Medium	High	Very high
Computational complexity	Low to medium	High	Medium (optimized)
Ability to capture context	Limited	High	High (with local optimization)
Scalability to high resolution	Limited	Limited (without optimization)	High
Potential in watermarking	Well verified but limited	High (based on previous research)	Very high

**Table 5:** Comparison of diffusion models and generative networks (GANs) in the context of watermarking.

Feature	GAN	Diffusion Model
Training stability	Low (frequent convergence problems)	High
Quality of generated images	High	Very high
Generation time	Relatively short	Longer (if no optimization methods were used)
Resistance to attacks	Medium	High
Computational complexity	Medium	High
Possibility of embedding in latent space	Limited	Yes
Potential in watermarking	Well verified but limited	High (based on previous research)

## Data Availability

No new data were created in this study. This survey analyzes and reviews publicly available datasets commonly used in image watermarking research, including ImageNet, COCO, CIFAR-10/100, Pascal VOC, BOSSBase, DIV2K, Flickr2K, LAION-5B, CIFAKE, ArtiFact, ImagiNet, NIH Chest X-ray, EuroSAT, and LFW. All datasets referenced in this article are openly accessible through their respective repositories as cited in the manuscript. Detailed dataset descriptions, resolutions, and usage contexts are provided in Table 3.

## Abbreviations

The following abbreviations are used in this manuscript:

CNN	Convolutional Neural Network
DCT	Discrete Cosine Transform
DFT	Discrete Fourier Transform
DWT	Discrete Wavelet Transform
GAN	Generative Adversarial Network
IoT	Internet of Things
PSNR	Peak Signal-to-Noise Ratio
SSIM	Structural Similarity Index
ViT	Vision Transformer
VIF	Visual Information Fidelity
